# Tungsten Nanoparticles Accelerate Polysulfides Conversion: A Viable Route toward Stable Room‐Temperature Sodium–Sulfur Batteries

**DOI:** 10.1002/advs.202105544

**Published:** 2022-02-08

**Authors:** Yuping Liu, Shuangying Ma, Marina Rosebrock, Pascal Rusch, Yvo Barnscheidt, Chuanqiang Wu, Pengfei Nan, Frederik Bettels, Zhihua Lin, Taoran Li, Binghui Ge, Nadja C. Bigall, Herbert Pfnür, Fei Ding, Chaofeng Zhang, Lin Zhang

**Affiliations:** ^1^ Institute of Solid State Physics Leibniz University Hannover Hannover 30167 Germany; ^2^ Laboratory of Nano and Quantum Engineering (LNQE) Leibniz University Hannover Hannover 30167 Germany; ^3^ Institute for Advanced Study Chengdu University Chengdu 610100 P. R. China; ^4^ SPEC CEA CNRS Université Paris‐Saclay CEA Saclay Cedex Gif‐sur‐Yvette 91191 France; ^5^ Institute of Physical Chemistry and Electrochemistry Leibniz University Hannover Hannover 30167 Germany; ^6^ Institute of Electronic Materials and Devices Leibniz University Hannover Hannover 30167 Germany; ^7^ Information Materials and Intelligent Sensing Laboratory of Anhui Province Key Laboratory of Structure and Functional Regulation of Hybrid Materials of Ministry of Education Institutes of Physical Science and Information Technology Anhui University Hefei 230601 China; ^8^ Cluster of Excellence PhoenixD Leibniz University Hannover Hannover 30167 Germany

**Keywords:** electrocatalyst, kinetics, large‐scale energy storage, room‐temperature sodium‐sulfur batteries, tungsten nanoparticles

## Abstract

Room‐temperature sodium–sulfur (RT Na–S) batteries are arousing great interest in recent years. Their practical applications, however, are hindered by several intrinsic problems, such as the sluggish kinetic, shuttle effect, and the incomplete conversion of sodium polysulfides (NaPSs). Here a sulfur host material that is based on tungsten nanoparticles embedded in nitrogen‐doped graphene is reported. The incorporation of tungsten nanoparticles significantly accelerates the polysulfides conversion (especially the reduction of Na_2_S_4_ to Na_2_S, which contributes to 75% of the full capacity) and completely suppresses the shuttle effect, en route to a fully reversible reaction of NaPSs. With a host weight ratio of only 9.1% (about 3–6 times lower than that in recent reports), the cathode shows unprecedented electrochemical performances even at high sulfur mass loadings. The experimental findings, which are corroborated by the first‐principles calculations, highlight the so far unexplored role of tungsten nanoparticles in sulfur hosts, thus pointing to a viable route toward stable Na–S batteries at room temperatures.

## Graphical abstract

A sulfur host material based on tungsten nanoparticles (with an average size of about 1.5 nm) embedded in nitrogen‐doped graphene is demonstrated (W@N—G), when used in room‐temperature sodium–sulfur batteries, the W@N—G can significantly accelerate the polysulfides conversion and completely suppress the shuttle effect, thus contributing to the unprecedented specific capacity, rate capability, and cycling stability.

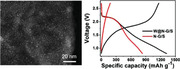

## 1 Introduction

Rechargeable batteries are a key enabler for the low‐carbon economy, and a tenfold increase of the annual battery capacity production is expected by the year 2030.^[^ ^1^ ^]^ Lithium‐ion batteries (LIBs) have been investigated over the decades,^[^ ^2^ ^]^ but they may not meet this rising requirement due to the rare and uneven geographical distribution of lithium.^[^ ^3^ ^]^ Na–S batteries are therefore attracting great interest in recent years, owing to the natural abundance of both sodium and sulfur, and to their high theoretical energy density of 1274 Wh kg^−1^.^[^ ^4^ ^]^ The construction of high‐performance Na–S batteries can learn from the Li–S battery studies, thanks to the similar conversion mechanisms in these two kinds of batteries.^[^ ^5^ ^]^ In fact, the molten sodium‐based high temperature (HR) Na–S batteries have been commercially available since the 1980s, but they have serious safety hazards and high operating costs.^[^ ^6^ ^]^ Besides, HR Na–S batteries with Na_2_S_3_ as the final discharge product only deliver about a third of the sulfur's theoretical capacity.^[^ ^6a^ ^]^

In light of these challenges, the research interest is moving toward the development of RT Na–S batteries. This is because the complete conversion from sulfur to sodium sulfide Na_2_S (i.e., the full capacity) can be achieved *potentially* via the electrochemical reaction 2Na + S → Na_2_S. However, RT Na–S batteries face their own set of challenges.^[^ ^6^, ^7^ ^]^ The insulating nature of sulfur limits its utilization and the electrochemical reaction kinetics.^[^ ^8^ ^]^ And the shuttle effect of the NaPSs results in the low specific capacity, insufficient Coulombic efficiency (CE), and poor cycle life.^[^ ^9^ ^]^ Tremendous efforts have been devoted to addressing these issues, such as using separator coatings^[^ ^10^ ^]^ and solid‐state electrolytes.^[^ ^4a^ ^]^ The sulfur host also plays a crucial role, as shown by numerous works in recent years.^[^ ^8^, ^9^, ^11^ ^]^ Carbon nanostructure hosts have good electrical conductivity and porous structures, but most of them are nonpolar and therefore do not have an effective interaction with the polar NaPSs.^[^ ^12^ ^]^ To solve this issue, polar sulfur hosts were reported recently. However, the weight ratio of these newly developed hosts is quite high and can be up to 40%,^[^ ^9^, ^13^ ^]^ thus limiting the achievable energy densities. Therefore, an ideal sulfur host should have strong chemisorption to the sulfur species, very efficient electrocatalytic properties to accelerate the conversion of NaPSs, and last but not least, a low weight ratio in the cathode.

In this work, we report a new type of sulfur host that can meet all these stringent requirements. Monodisperse tungsten nanoparticles embedded in nitrogen‐doped graphene (W@N‐G) were synthesized via a simple pyrolysis reaction.^[^ ^14^ ^]^ Their facile fabrication process (see Experimental Section) holds a strong potential for practical applications. When used in RT Na–S batteries, the W@N‐G/S composite cathode exhibited *extraordinary* adsorption and electrocatalytic characteristics. The experimental results show a strong interaction between the W@N‐G host and NaPSs, which is further corroborated by the first‐principles calculations. The incorporation of W increases the binding energies (between the host and NaPSs) by nearly 200% to 300%, thus contributing to the unprecedented specific capacity, rate capability, and cycling stability. It is worth emphasizing that the weight ratio of W@N‐G is only 9.1% in the cathode, being 3 to 6 times lower than that in recent reports on Na–S batteries. Our work provides the first insight into the strong interaction between W nanoparticle catalysts and NaPSs, thus offering a viable route toward the realization of stable Na–S batteries at room temperatures.

## 2 Results and Discussion

### 2.1 Material Synthesis and Characterization

**Figure 1:**
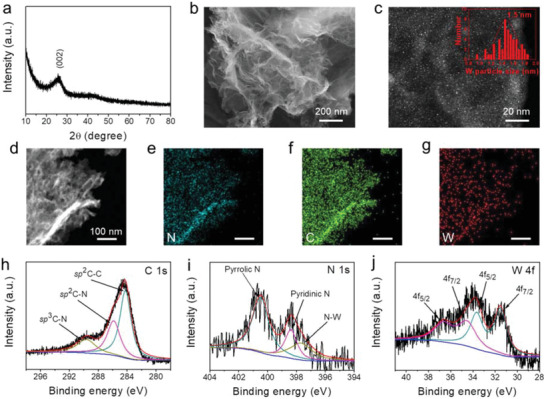
Morphological and structural characterizations of the W@N—G nanosheets. a) XRD. b) SEM. c) STEM image (insert is the size distribution of W nanoparticles). d–g) HAADF‐STEM and the corresponding element mapping images. XPS analysis of the h) C 1s, i) N 1s, and j) W 4f spectrum.

**Figure 2:**
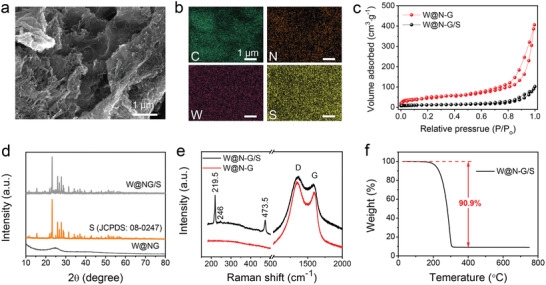
Characterization of the W@N—G/S cathode. a,b) SEM and corresponding elemental mapping images of W@N—G/S cathode. c) Nitrogen adsorption‐desorption isotherms of W@N‐G and W@N‐G/S. d) XRD patterns of W@N—G, S, and W@N—G/S. e) Raman spectra of W@N—G and W@N—G/S. f) TGA curve of W@N—G/S cathode under N_2_ atmosphere.

W@N‐G nanosheets were fabricated by the in situ pyrolysis (see Experimental Section).^[^ ^14^ ^]^ X‐ray diffraction (XRD) was employed to confirm the crystal phases of the W@N‐G nanosheets, and the main diffraction peak at about 25° can be assigned to the (002) plane of graphite (**Figure** **1**a).^[^ ^14a^ ^]^ The absence of W‐based patterns indicates the highly dispersive nature of W nanoparticles in the N‐doped graphene, which is further corroborated by HAADF‐STEM studies. The structure of the W@N‐G nanosheets was further investigated by Raman spectroscopy. Two characteristic carbon peaks were observed, which correspond to the defect‐related D band at ≈1358 cm^−1^ and the in‐plane *E*_2_ *_g_* phonon mode (G band) at ≈1595 cm^−1^, respectively (**Figure** **2**e).^[^ ^15^ ^]^

The scanning electron microscope (SEM) reveals the graphene‐like nanosheet morphology of the W@N‐G, and no obvious aggregation can be observed (Figure 1b). The Brunauer–Emmett–Teller study shows a specific surface area of 466 m^2^ g^−1^ (Figure 2c). This large surface area alleviates the dissolution of NaPSs intermediates via chemisorption, and effectively catalyzes the conversion of NaPSs, both of which help to boost the electrochemical performances of RT Na–S batteries. Scanning transmission electron microscopy (STEM) was also performed, see Figure 1c and Figure S1, Supporting Information. W nanoparticles, with an average size of about 1.5 nm, are uniformly distributed on the nanosheets. The smaller size of the catalysts will provide more active sites for the NaPSs, however, the size reduction maybe results in the formation of metastable state metal nanoparticles, thus the nanoparticles should be controlled in an appreciated size. Moreover, the elemental mappings by using energy dispersive spectroscopy illustrate the homogeneous distribution of N, C, and W throughout the nanosheet (Figure 1d–g). For comparison, the reference N‐G sample also shows the graphene‐like nanosheet morphology, similar to that of W@N‐G but without the presence of W nanoparticles (Figure S2a,b, Supporting Information).

To investigate the electronic structure of the C, N, and W species in the W@N‐G, high‐resolution X‐ray photoelectron spectroscopy (XPS) was performed. The C 1s scan reveals three peaks after deconvolution. The main peak at 284.3 eV can be assigned to the sp^2^C—C bond of graphene, while the other two peaks at 286 and 288 eV are from the sp^2^C—N and sp^3^C—N bonds, respectively (Figure 1h). These peaks originate from the N‐doping in the graphene.^[^ ^14^, ^16^ ^]^ The N 1s spectra exhibit three peaks—the peak with the lowest binding energy is assigned to the W‐N species,^[^ ^17^ ^]^ while the other two are associated with the pyrrolic N and pyridinic N, respectively (Figure 1i).^[^ ^18^ ^]^ W is coordinated with N, which achieved a cationic coordination environment and showed different oxidation states, the W 4f spectra show two characteristic doublets at 36.665/34.515 eV and 33.665/31.515 eV with a separation of about 2.15 eV, which are associated with the W^2+^ and W^5+^ oxidation states (Figure 1j).^[^ ^17^, ^19^ ^]^ From the XPS analysis, the W atom ratio in the W@N‐G can be calculated to be about 5.4 atom %. For comparison, the XPS spectra of the N—G sample are shown in Figure S2c‐d, where the C 1s and N 1s scans are similar to that of the W@N‐G sample but without the presence of the W—N bond.

Elemental sulfur was then impregnated in the W@N‐G host through a melt‐diffusion process to obtain the S‐loaded W@N‐G (W@N‐G/S) cathode. Figure 2a shows a representative SEM image, where the rough and rugged surfaces can be observed after the S‐loading. The corresponding elemental mapping reveals the homogenous distribution of C, N, W, and S throughout the nanosheets (Figure 2b). Due to the successful infilling/adsorption of sulfur, the W@N‐G/S cathode shows a surface area of 294 m^2^ g^−1^ (Figure 2c), which is reduced by 37% compared to the pure W@N‐G nanosheets.

Figure 2d shows the XRD patterns of the W@N‐G, sublimed sulfur, and W@N‐G/S composite. The XRD pattern of W@N‐G/S is the composition of the characteristic peaks of both W@N‐G and elemental S (JCPDS: 08‐0247),^[^ ^20^ ^]^ indicating the stable crystallinity of S after being loaded on the W@N‐G nanosheets. Raman spectroscopy was also performed, and the peaks at 219.5, 246.0, and 473.5 cm^−1^ can be assigned to the impregnated S (Figure 2e).^[^ ^21^ ^]^ It is interesting to mention that the intensity ratio *I*_D_/*I*_G_ is about 1.17 for W@N‐G/S, indicating rich defects in the graphene.^[^ ^22^ ^]^ According to recent studies, this would result in an improved catalytic/chemisorption effect on the NaPSs.^[^ ^13^ ^]^

Here we discuss another important parameter when designing Na–S batteries. Many of the host/catalyst materials can adsorb polysulfides and catalyze their conversions, but they do not contribute to the capacity. In order to achieve a high energy density, the weight ratio of the catalyst should be strictly controlled when constructing S cathodes. In this work, the thermogravimetric analysis (TGA) shows that the W@N‐G catalyst is only 9.1 wt% in the cathode (Figure 2f), which is significantly (about 3 to 6 times) lower than that in recent reports on Na–S batteries (Table S1, Supporting Information). For comparison, the TGA curve of the N‐G/S cathode is shown in Figure S3, Supporting Information.

### 2.2 Reaction Mechanism

**Figure 3:**
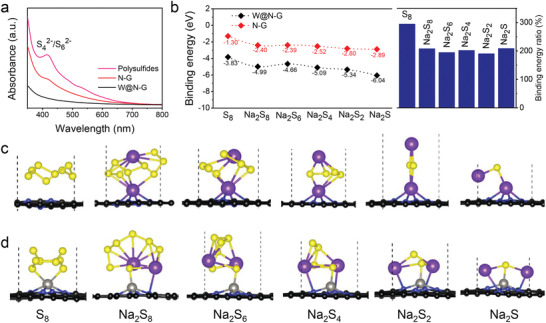
Illustrations of the interactions between NaPSs and the N—G or W@N—G nanosheets, respectively. a) UV–vis spectra of Na_2_S_6_ solution after exposure to N—G or W@N—G. b) Binding energies between NaPSs and W@N—G (black) or N—G (red), and their ratios (right). The optimized adsorption conformations of NaPSs on c) N—G and d) W@N—G and. The black, yellow, purple, pink, and grey balls represent C, S, Na, N, and W atoms, respectively.

To elucidate the interaction between the W@N‐G nanosheets and the NaPSs, UV–vis spectroscopy was performed. As shown in **Figure** **3**a, the solution added with N‐G shows the characteristic absorption peak of NaPSs in the range of 400–450 nm.^[^ ^23^ ^]^ The same peak disappears completely in the spectrum for the solution to which added with W@N‐G, indicating the strong adsorption of NaPSs due to the existence of W. The strong interaction between the W@N‐G host and NaPSs was further demonstrated by employing the adsorption experiments (Figure S4, Supporting Information). The same amount of W@N—G or N—G was added into the NaPSs solution. As shown in Figure S4, Supporting Information, the solution color changed from dark brown to colorless or to light brown, after adding W@N—G or N—G, respectively. These results agree very well with the UV–vis spectroscopy results in Figure 3a.

To gain a deeper insight into the chemical interaction between NaPSs and W@N—G (with N/G used as a reference), the first‐principles calculations were performed. Here we investigate the binding energies, which are an important parameter to demonstrate the immobilization ability of the sulfur host materials. In our model, each W atom locates at out of the graphene plane and bonds with the adjacent four N atoms to form a pyramid structure. The optimized structures of W@N—G and N—G are shown in Figure S5, Supporting Information. Although the graphene shows the van der Waals interaction with NaPSs,^[^ ^24^ ^]^ however, after the N‐doped and introduction of W, the Na and S binds with the N and W on the W@N‐G, respectively, and shows significantly higher binding energy with NaPSs, which apparently stems from the contribution of chemical interactions. The calculated binding energies of S_8_, Na_2_S_8_, Na_2_S_6_, Na_2_S_4_, Na_2_S_2_, and Na_2_S for the N‐G structure are −1.30, −2.40, −2.39, −2.52, −2.80, and −2.89 eV, respectively. Here, a more negative value indicates an energetically more favorable bonding to the substrate. The W@N—G structure shows greatly improved adsorption capability of −3.83, −4.99, −4.66, −5.09, −5.34, and −6.04 eV, respectively. The ratio of the binding energies between these two cases (i.e., with and without the W atoms) are about 200% to 300% (Figure 3b), strongly confirming the improved confinement of NaPSs and therefore the efficient suppression of the shuttling effect. Interestingly, according to the recent study, the decomposition barriers of NaPSs are correlated with the adsorption energies—higher binding energies result in a lower decomposition energy barrier.^[^ ^24^ ^]^ Therefore, the W@N‐G can also accelerate the rate of oxidative decomposition Na−S bonds, which is beneficial to high‐rate capability and avoids the loss of active material as Na_2_S precipitate. The binding energies of the optimized orientation of NaPS_S_ on the W@N‐G and N‐G are compared in Figure 3c,d, and Figure S6, Supporting Information. In addition, the partial density of states (PDOS) change of W@N‐G near Fermi level (after the adsorption of NaPSs), which further corroborates to the charge transfer from the NaPSs to the W@N‐G substrate during the electrochemical process (Figure S7, Supporting Information).

Furthermore, the glass fiber separator was significantly colored due to the shuttle effect in the N‐G/S cathode (after 3 cycles), which was not observed in the case of the W@N‐G/S electrode (Figure S8, Supporting Information). This is corroborated by the observation of many holes in the N—G/S cathode, which is due to the dissolution and shuttling of NaPSs in the electrolyte. The morphology of the W@N—G/S cathode, on the contrary, has no obvious change after cycling—a strong evidence of the improved electrochemical adsorption between the W@N—G host and NaPSs (Figure S9, Supporting Information).

**Figure 4:**
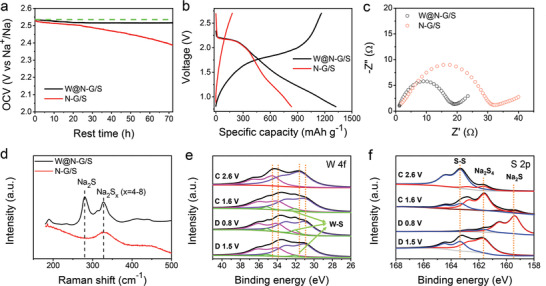
Electrochemical adsorption/catalytic mechanism of W@N—G toward NaPSs. a) Self‐discharge behavior. b) The first charge/discharge curves of W@N—G/S and N‐G/S cells (0.2 C). c) EIS spectra of W@N—G/S and N—G/S cells after three cycles. d) Raman spectra of W@N—G/S and N—G/S cathodes after discharged to 0.8 V. Ex situ XPS spectra of W@N‐G/S cathode at different states, e) W 4f, f) S 2p.

The self‐discharging behavior of the cells with W@N—G/S and N—G/S cathodes was measured while resting at room temperature. As shown in **Figure** **4**a, an obvious voltage drop (≈6% in 72 h) of the N—G/S cathode can be observed, which is evidence of a serious shuttle in this battery. The W@N—G/S cathode, on the other hand, only shows a slight voltage drop (≈0.6%) in the first 20 h and then keeps stable for the rest 52 h, suggesting a strong suppression of the shuttle effect of NaPSs.^[^ ^25^ ^]^

The galvanostatic charge/discharge profiles of these two cathodes are displayed in Figure 4b (see also Figure S10, Supporting Information). The plateau at ≈2.2 V in the initial discharge originates from the irreversible reaction of surface S_8_, followed by a long slope that corresponds to the transformations to the soluble Na_2_S_4_, and then to the insoluble Na_2_S.^[^ ^9^, ^13^ ^]^ The W@N‐G/S cell delivers a higher initial discharge capacity than the N‐G/S cell when fully discharged to 0.8 V (1350 mAh g^−1^ and 820 mAh g^−1^, respectively). Another major difference occurs in the subsequent cycles. The W@N‐G/S cell shows a reversible specific capacity of about 1000 mAh g^−1^, more than 3 times larger than that of the N—G/S cell (280 mAh g^−1^). Figure 4c shows the impedance test after three cycles, further supporting this conclusion. The impedance of the W@N‐G/S cell reaches about 18 Ω, while the impedance value of the N—G/S cell is about 32 Ω. This is evidence of the enhanced conductivity after the incorporation of W.^[^ ^26^ ^]^ Raman spectroscopy was performed for both of the W@N—G/S and N—G/S cathodes after being discharged to 0.8 V, and the characteristic peak of Na_2_S can be only observed in the case of W@N—G/S (Figure 4d).

The ex situ XPS was performed to reveal the corresponding sodiation/desodiation mechanism. Figure 4e shows the changes of W 4f valence state of W@N—G during the charging/discharging. Because the electrons are transferred from W to NaPSs, the W 4f spectra are shifted to the lower binding energy side. The two new components at 35.18 and 32.07 eV can be assigned to the W—S bonds, indicating the strong chemical interaction between W@N‐G and NaPSs. Importantly, when the cell was charged back to 2.6 V, the peaks of W 4f were transformed back to the pristine binding energy, and the W‐S bonds were almost disappeared. For the S 2p spectra (Figure 4f), the peaks at 162.86 and 161.7 eV were detected at the discharging voltage of 1.5 V, corresponding to the existences of Na_2_S_6_/Na_2_S_4_.^[^ ^27^ ^]^ At 0.8 V, these peaks downshifted to 160.56 and 159.4 eV, illustrating the transformation to Na_2_S_2_/Na_2_S.^[^ ^27^, ^28^ ^]^ Similar peaks were not observed for the N‐G/S cathode after being discharged to 0.8 V, demonstrating that the N‐G host is incapable of converting the soluble NaPSs to the insoluble Na_2_S (Figure S11, Supporting Information). When the discharging voltage returns to 2.6 V, NaPSs is fully reduced to sulfur.

These results are highly consistent with the aforementioned electrochemical performances, and confirm the extraordinary role of W nanoparticles in promoting the conversion of the NaPSs and maintaining the cycling stability of the cells. This provides a viable route toward RT Na–S batteries, considering that 75% of the capacity is contributed by the conversion between the soluble NaPSs and the insoluble Na_2_S.^[^ ^27^ ^]^

### 2.3 Electrochemical Performance

**Figure 5:**
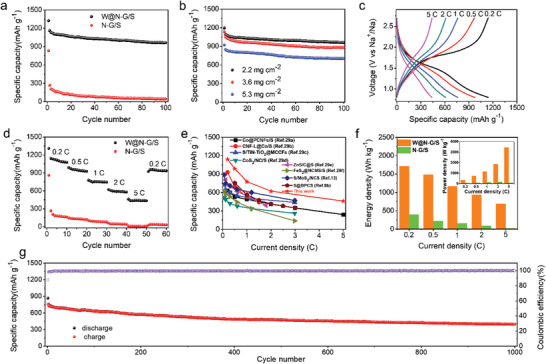
Electrochemical performance of RT Na–S batteries. a) Cycling performance of W@N—G/S and N—G/S cathodes (current density: 0.2 C, S mass loading: 1 mg cm^−2^). b) Cycling performance of W@N—G/S cathodes with different S mass loadings (current density: 0.2 C). c) Charge–discharge curves at various current densities of the W@N—G/S cathode. d) Rate performance of the W@N—G/S and N—G/S cathodes. e) Comparison of this work with previously reported RT‐Na/S batteries regarding the rate capability. f) Energy density and power density are based on the electrode materials. g) Long‐term cycling performance at 1 C (1 mg cm^−2^).

Due to the incorporation of W nanoparticles, the W@N—G/S cathode delivers not only a higher specific capacity, but also a much better cycling stability (**Figure** **5**a). The discharge capacity was 1160 mAh g^−1^ in the second cycle and still reached 962 mAh g^−1^ after 100 cycles. In contrast, the N—G/S cathode delivered a discharge capacity of 267 mAh g^−1^ in the second cycle, and only remained 43 mAh g^−1^ after 100 cycles. In order to evaluate the potential of W@N—G host in practical applications, higher S mass loadings were further investigated. Here, the W@N—G/S cathodes with 3.6 and 5.3 mg cm^−2^ were measured at 0.2 C (Figure 5b). A discharge capacity of 1050 mAh g^−1^ was obtained in the second cycle and reached 883 mAh g^−1^ after 100 cycles (3.6 mg cm^−2^). The electrode with 5.3 mg cm^−2^ S mass loading showed a lower initial discharge capacity, but still much higher than that of the N—G/S cathode and also with an impressive cycling stability.

The rate capabilities of the W@N‐G/S cathode were measured under a current density from 0.2 to 5 C (Figure 5c,d). The reversible capacities are 1142, 988, 777, 622, and 461 mAh g^−1^ at the rate of 0.2, 0.5, 1, 2, and 5 C, respectively. Even at 5 C, the charge/discharge curves still exhibit a plateau, suggesting the fast sulfur redox reaction kinetics due to the W@N—G catalyst. The discharge capacity recovered to a high value after switching the current density to 0.2 C, confirming a good reversibility. The N—G/S cathode, on the other hand, showed extremely poor rate capabilities, and only delivered 14 mAh g^−1^ of capacity under the large current density of 5 C.

To our knowledge, this rate performance is arguably the best for RT Na–S batteries reported so far (Figure 5e).^[^ ^9^, ^13^, ^29^ ^]^ This also gives rise to the much higher gravimetric energy/power densities compared to that of the N‐G/S cathode (Figure 5f). The cycling stability of the W@N—G/S cathode is shown in Figure 4g, and a high reversible capacity of 398 mAh g^−1^ can be reached even after 1000 cycles, corresponding to a low capacity decay of 0.036% per cycle. For practical applications, mass loading, current density, and cycling stability are crucial factors. Thus, the W@N‐G/S cathode with 5 mg cm^−1^ S loading was also evaluated under 1 C, and the result is quite satisfactory (Figure S12, Supporting Information).

## 3 Conclusion

In summary, we report a new type of sulfur host that is based on tungsten nanoparticles embedded in nitrogen‐doped graphene. The incorporation of tungsten nanoparticles drastically improves the chemisorption and electrocatalization of NaPSs in RT Na–S batteries. With a host weight ratio of only 9.1% (about 3–6 times lower than that in recent reports), the cathode shows a strong affinity to NaPSs, complete suppression of the shuttle effect, and enhanced kinetic conversion of the NaPSs in the electrochemical process. As a result, the constructed batteries exhibit high specific capacity (up to 1160 mAh g^−1^), excellent rate capability (461 mAh g^−1^ at 5 C), and superior cycling performance (0.036% decay per cycle for 1000 cycles). The incorporation of tungsten nanoparticles also allows the much‐improved battery performances when subjected to the stringent conditions (e.g., 5 mg cm^−1^ sulfur loading at 1 C). The constructed battery has arguably the best rate performances among its peers. The mechanism behind the experimental findings is clearly disclosed by the first‐principles calculations. Our work highlights the unprecedented role of tungsten‐based sulfur host in adsorbing and catalyzing the NaPSs, and, in more general terms, points to a viable route of designing practical room temperature Na–S batteries with the transition‐metal nanoparticles.

## Conflict of Interest

The authors declare no conflict of interest.

## Supporting information

Supporting Information

## Data Availability

Research Data are not shared.
