# Supplementary material for: Tungsten Nanoparticles Accelerate Polysulfides Conversion: A Viable Route toward Stable Room‐Temperature Sodium–Sulfur Batteries
Source: Adv Sci (Weinh). 2022 Feb 8;9(11):2105544. doi: 10.1002/advs.202105544 (PMC9008787; doi:10.1002/advs.202105544)
Supplement: Supplementary file 1 — Supporting Information [file ADVS-9-2105544-s001.pdf]

ADVANCED SCIENCE
Open Access

Supporting Information

for *Adv. Sci.*, DOI: 10.1002/advs.202105544

Tungsten Nanoparticles Accelerate Polysulfides Conversion: A Viable Route toward Stable Room-Temperature Sodium-Sulfur Batteries

*Yuping Liu, Shuangying Ma, Marina Rosebrock, Pascal Rusch, Yvo Barnscheidt, Chuanqiang Wu, Pengfei Nan, Frederik Bettels, Zhihua Lin, Taoran Li, Binghui Ge, Nadja C. Bigall, Herbert Pfnür, Fei Ding, Chaofeng Zhang,and Lin Zhang**

Supporting Information

Tungsten Nanoparticles Accelerate Polysulfides Conversion: A Viable Route toward Stable Room-Temperature Sodium-Sulfur Batteries

Yuping Liu,[1,2] Shuangying Ma,[3,4] Marina Rosebrock,[2,5] Pascal Rusch,[2,5] Yvo Barnscheidt,[6] Chuanqiang Wu,[7] Pengfei Nan,[7] Frederik Bettels,[1,2] Zhihua Lin,[1,2] Taoran Li,[1,2] Binghui Ge,[7] Nadja C. Bigall,[2,5,8] Herbert Pfnür,[1,2] Fei Ding,[1,2] Chaofeng Zhang,[7] and Lin Zhang[1,2]*

*Corresponding Authors: lin.zhang@fkp.uni-hannover.de

Experimental Section

Materials: Glucose and dicyandiamide were purchased from Sigma-Aldrich. Ammonium tungsten oxide hydrate ($(NH_4)_6W_{12}O_{39}$•xH_2O) was purchased from Alfa-Aesar. All the chemical reagents were used without further purification.

Preparation of W@N-G and W@N-G/S: 0.25 g glucose and 5 g dicyandiamide were dissolved into 250 mL deionized water, and then 2 mL $(NH_4)_6W_{12}O_{39}$•xH_2O salt solution (0.025 M) was added dropwise into the above solution under the protection of N_2. The mixture was stirred and heated at 80 ℃ for 2 h. After cooling down and freeze-drying, the precursor was annealed at 600 ℃ for 2 h and then rose to 900 ℃ for another 2 h (with a rate of 3 ℃/min). The S loading into/onto the W@N-G host is through a melt-diffusion process, firstly, mixing excess sulfur with W@N-G, and then heating at 155 °C under an Ar atmosphere for 12 h, finally, further heating at 200 °C for 2 h to obtain the W@N-G/S cathode. As a comparison, nitrogen-doped graphene (NG) was also prepared using the same method without the addition of $(NH_4)_6W_{12}O_{39}$•xH_2O salt solution.

Materials Characterization: XRD patterns were collected on a Bruker D8 Advance Discovery X-ray Diffractometer. Raman spectroscopy was carried out on the inVia Raman spectrometer from Renishaw with a HeNe laser (632.8 nm excitation wavelength). The morphology, microstructure, and composition of samples were characterized by emission scanning electron microscopy (FE-SEM, JEOL JSM-6700F) and transmission electron microscopy (FEI Talos F200X equipped with One View camera operated at 200 kV). XPS analysis was performed with a hemispherical analyzer of 100 mm radius (Leybold Heraeus). For the *ex-situ* XPS, the electrodes were measured after three cycles and opened the batteries in the glove box, then the electrode surfaces were cleaned carefully use the

pure PC/EC, after dried, the electrodes were covered in the XPS holder and transferred to the XPS chamber. Thermogravimetric analysis (Mettler Toledo TGA/DSC 3+) was conducted under the N_2 atmosphere by heating from RT to 750 ℃ at 5 ℃ min^{-1}. Ultraviolet/visible absorbance spectroscopy was performed in the spectral range of 200-800 nm using a Cary 5000 UV–vis variable wavelength spectrophotometer to evaluate the sodium NaPSs absorption capability of W@N-G and NG composite (the Na_2S_6 solution was synthesized by mixing Na_2S and sulfur in a stoichiometric ratio of 2:6 in DME (dimethoxyethane)).

The W Nanoparticles Size Statistical Analysis: The W nanoparticles size distribution was calculated by the Photoshop soft, briefly, enlarge the STEM image 100 times (Figure 2c), select 50 W nanoparticles (random) and measure the largest length of each nanoparticle, and finally calculate the size distribution.

Electrochemical Measurements: The working electrodes for the Na-S cells were fabricated by mixing the as-synthesized composites (W@N-G/S or NG/S), carbon black, and polyvinylidene difluoride (PVDF) with a weight ratio of 8: 1: 1 in N-methyl-2-pyrrolidone (NMP) to form a slurry, then uniformly pasted on the aluminum foil followed by drying under vacuum oven at 60 °C overnight. The Na-S cells were assembled with metallic sodium as the anode and the W@N-G/S (or NG/S) as the cathode by the CR2032 coin cell, glass fiber (Whatman GF/F) as the separator, and 1 M $NaClO_4$ in 1:1 (volume ratio) ethylene carbonate/propylene carbonate (PC/EC) with 3 wt. % fluoroethylene carbonate (FEC) additive as the electrolyte, and 5 μL mg^{-1} and 10 μL mg^{-1} electrolyte was used in low and high (> 3 mg cm^{-2}) sulfur loading electrodes, respectively. The assembly of all the Na-S cells was carried out in the Ar-filled glovebox (MBraun) with water and oxygen

concentrations less than 0.1 ppm. The electrochemical properties of the W@N-G/S and NG/S cathodes were investigated by the LAND CT 2001A charge/discharge system with a cut-off voltage range from 0.8 to 2.6 V (vs. Na/Na^+). The cyclic voltammetry (CV, at a scan rate of 0.1 mV s^{-1}) and electrochemical impedance spectroscopy (EIS) of the cells were conducted using Metrohm Auto-lab.

Computational Methods: Based on density functional theory, the first-principles calculations were performed using LCAO calculator as implemented in QuantumATK package to investigate the equilibrium configurations and adsorption energies of NaPSs (Na_2S_n (n=1, 2, 4, 6, 8)) and S_8 on N-doped graphene film.[1] The valence electrons and core interactions were described with PseudoDojo pseudopotentials. A generalized gradient approximation (GGA) proposed by Perdew, Burke, and Ernzerhof (PBE) was used to treat the exchange-correlation functional.[2] A density mesh cut-off of 120 Ha was used to ensure reliable accuracy. The van der Waals (vdW) correction was also considered by using a Grimme DFT-D3 dispersion term.[3] A 6 × 7 × 1 supercell of graphene was utilized to adsorb NaPSs. A vacuum layer of at least 20 Å perpendicular to the graphene film was applied to avoid the interaction between neighboring images. The first Brillouin zone was sampled using a 2 × 2 × 1 and 6 × 5 × 1 Monkhorst-Pack k-point scheme for structural optimization and adsorption energy calculations. All the structures were fully relaxed until the residual Hellmann-Feynman force on each atom is smaller than 0.01 eV$Å^{-1}$. The total energy convergence criterion was 1×10^{-6} eV. The adsorption energy was defined as:

$$E_{adsorption} = E_{polysulfides/substate} - E_{polysulfides} - E_{substrate} \qquad (1)$$

Where $E_{polysulfides/substate}$, $E_{polysulfides}$, and $E_{substrate}$ denote the total energies of the NaPSs molecule (Na_2S_n (n=1, 2, 4, 6, 8)) or S_8 adsorbed on the substrate (W@N-G or N-G), single PSs molecule and substrate, respectively.

Table S1. A list of the catalyst weight ratio in sulfur cathode and the electrochemical performances in the recently reported RT Na-S batteries.

Catalyst	Ratio	Initial capacity (2nd, mAh g^{-1})	Capacity retention	Rate performance	Reference
Co@PCNFs/S	**50%**	600 (0.5 C)	398 (after 600 cycles)	240 (5 C)	[4]
CNF-L@Co/S	**55%**	745 (0.5 C)	538 (after 150 cycles)	442.7 (1.5 C)	[5]
S/TiN-TiO_2@MCCFs	**43.1%**	1150 (0.1 A g^{-1})	640.4 (after 100 cycles)	440.2 (5 A g^{-1})	[6]
CoS_2/NC/S	**49.3%**	700 (0.1 A g^{-1})	488 (after 100 cycles)	262 (5 A g^{-1})	[7]
ZnS/C@S	**38%**	1070 (0.1 A g^{-1})	1082 (after 100 cycles)	390 (3 A g^{-1})	[8]
FeS_2@NCMS/S	**34.5%**	760 (0.1 A g^{-1})	524 (after 300 cycles)	337 (5 C)	[9]
S/MoS_2/NCS	**56.2%**	970 (0.5 A g^{-1})	711.6 (after 200 cycles)	470.7 (5 A g^{-1})	[10]
S@BPCS	**30%**	755 (0.5 C)	701 (after 350 cycles)	349 (3 C)	[11]
W@N-G	**9.1%**	1160 (0.2 C,)	962 (after 100 cycles)	461 (5 C)	This work

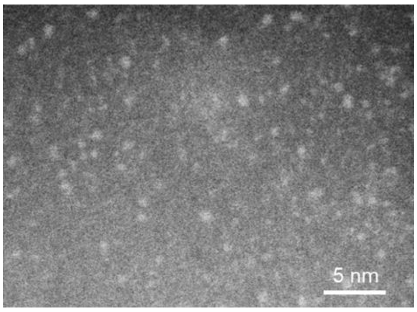

Figure S1. STEM of the W@N-G.

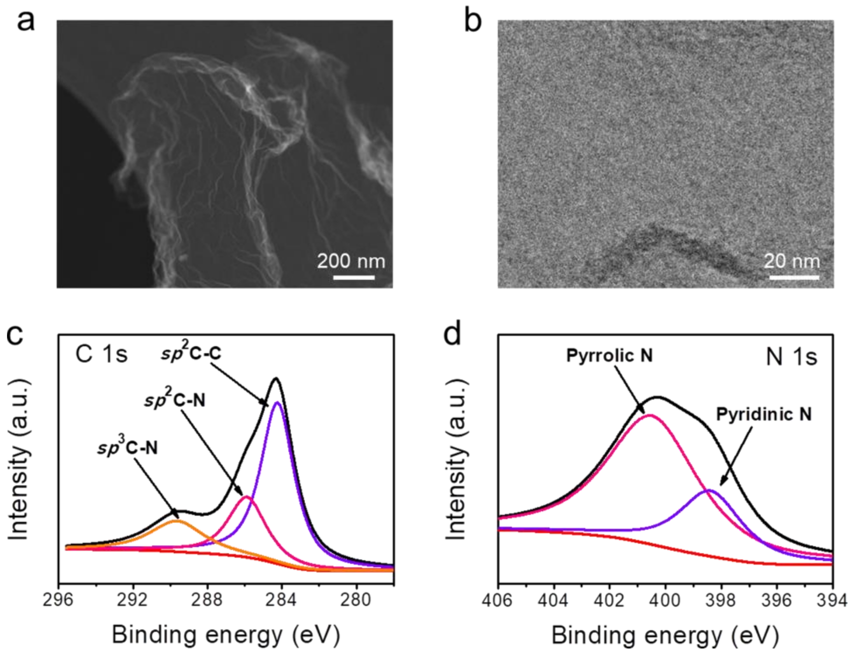

Figure S2. Morphological and structural characterizations of the N-G nanosheets. (a) STEM, and (b) HRTEM. XPS analysis of the (c) C 1s, and (d) N 1s spectrum.

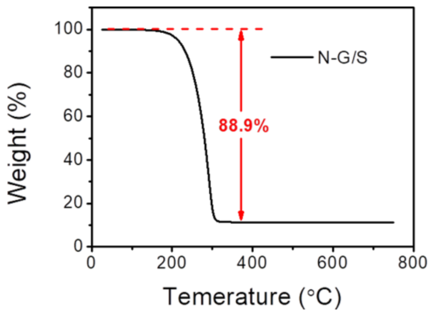

Figure S3. TGA curve of N-G/S cathode under N_2 atmosphere.

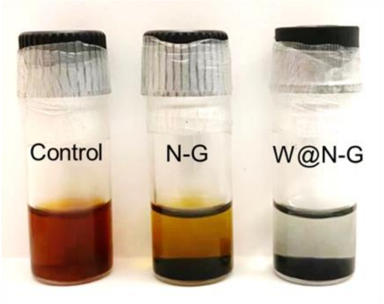

Figure S4. Photos showing the colour changes of Na_2S_6 solution after the exposure to N-G or W@N-G.

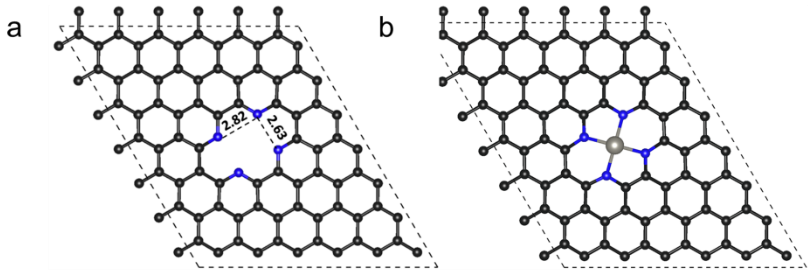

Figure S5. Structures of (a) N-G and (b)W@N-G hosts used in first-principles calculations.

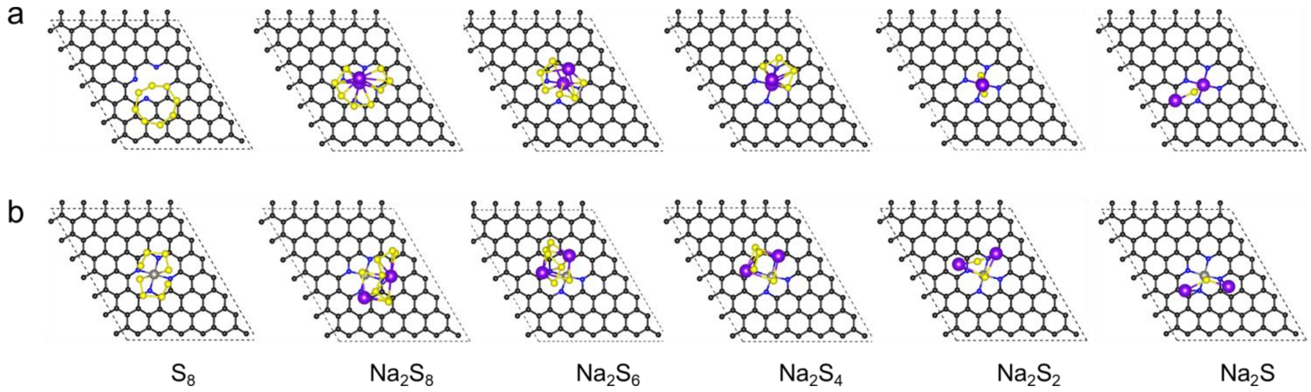

Figure S6. Top view of the optimized adsorption conformations of intermediate species on (a) N-G, and (b) W@N-G.

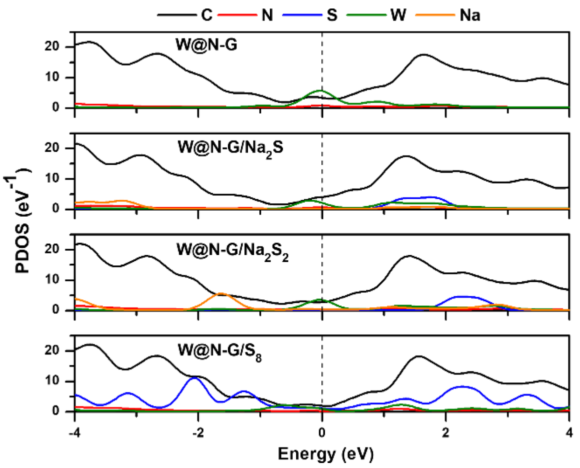

Figure S7. The electronic properties of W@N-G with and without the adsorption of NaPSs.

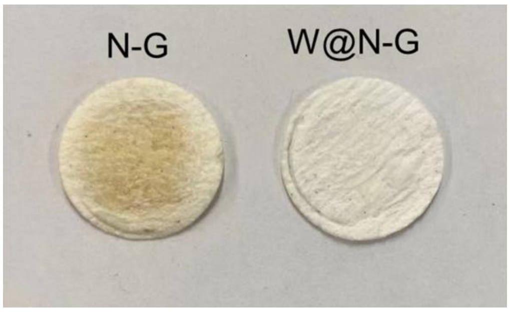

Figure S8. Photos showing the glass fiber separators after three cycles.

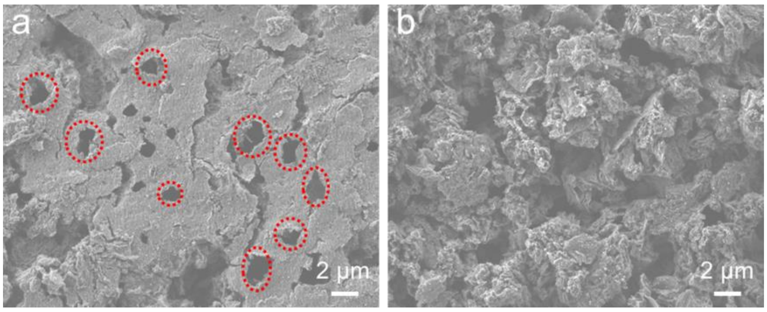

Figure S9. SEM of (b) N-G/S and (b) W@N-G/S cathodes after three cycles.

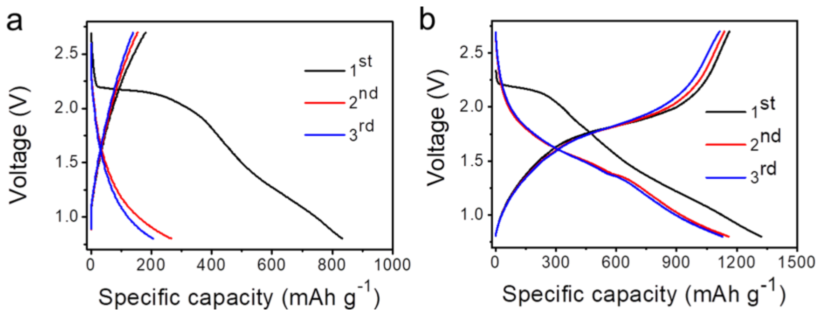

Figure S10. First three charge/discharge curves of (a) N-G/S and (b) W@N-G/S cathodes.

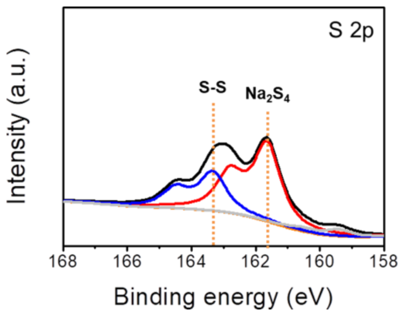

Figure S11. *Ex-situ* XPS spectra of N-G/S cathode after discharged to 0.8 V (after three cycles).

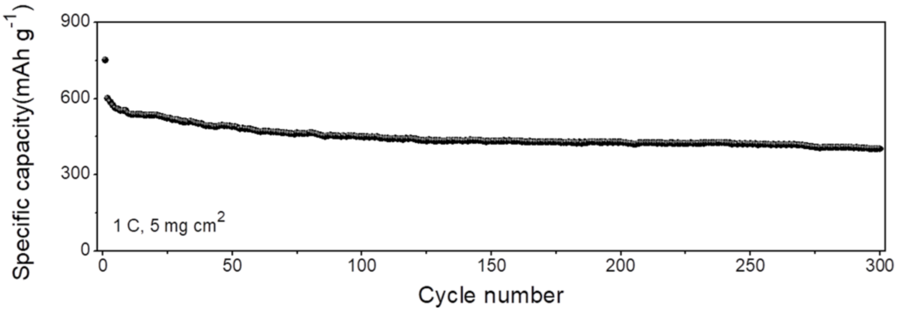

Figure S12. Cycling performance of W@N-G/S cathode with high S mass loading under large current.

Reference

[1] S. Smidstrup, T. Markussen, P. Vancraeyveld, J. Wellendorff, J. Schneider, T. Gunst, B. Verstichel, D. Stradi, P. A. Khomyakov, U. G. Vej-Hansen, M. E. Lee, S. T. Chill, F. Rasmussen, G. Penazzi, F. Corsetti, A. Ojanpera, K. Jensen, M. L. N. Palsgaard, U. Martinez, A. Blom, M. Brandbyge, K. Stokbro, *J. Phys-Condens. Mat.* **2020**, *32*, 015901.

[2] J. P. Perdew, K. Burke, M. Ernzerhof, *Phys. Rev. Lett.* **1996**, *77*, 3865.

[3] S. Grimme, *J. Comput. Chem.* **2006**, *27*, 1787.

[4] H. L. Yang, S. Zhou, B. W. Zhang, S. Q. Chu, H. P. Guo, Q. F. Gu, H. W. Liu, Y. J. Lei, K. Konstantinov, Y. X. Wang, S. L. Chou, H. K. Liu, S. X. Dou, *Adv. Funct. Mater.* **2021**, 2102280.

[5] W. Y. Du, K. Q. Shen, Y. R. Qi, W. Gao, M. L. Tao, G. Y. Du, S. J. Bao, M. Y. Chen, Y. M. Chen, M. W. Xu, *Nano-Micro Letters* **2021**, *13*, 1.

[6] X. Ye, J. F. Ruan, Y. P. Pang, J. H. Yang, Y. F. Liu, Y. Z. Huang, S. Y. Zheng, *ACS Nano* **2021**, *15*, 5639.

[7] F. P. Xiao, H. K. Wang, T. H. Yao, X. Zhao, X. M. Yang, D. Y. W. Yu, A. L. Rogach, *ACS Appl. Mater. Inter.* **2021**, *13*, 27735.

[8] H. W. Liu, W. H. Lai, Y. R. Liang, X. Liang, Z. C. Yan, H. L. Yang, Y. J. Lei, P. Wei, S. Zhou, Q. F. Gu, S. L. Chou, H. K. Liu, S. X. Dou, Y. X. Wang, *J. Mater. Chem. A* **2021**, *9*, 566.

[9] Z. C. Yan, Y. R. Liang, J. Xiao, W. H. Lai, W. L. Wang, Q. B. Xia, Y. X. Wang, Q. F. Gu, H. M. Lu, S. L. Chou, Y. Liu, H. K. Liu, S. X. Dou, *Adv. Mater.* **2020**, *32*, 2100229.

[10] Y. X. Wang, Y. Y. Lai, J. Chu, Z. C. Yan, Y. X. Wang, S. L. Chou, H. K. Liu, S. X. Dou, X. P. Ai, H. X. Yang, Y. L. Cao, *Adv. Mater.* **2021**, *33*, 2100229.

[11] M. K. Aslam, I. D. Seymour, N. Katyal, S. Li, T. T. Yang, S. J. Bao, G. Henkelman, M. W. Xu, *Nat. Commun.* **2020**, *11*, 1.
